# Non-clinical safety evaluation of salvianolic acid A: acute, 4-week intravenous toxicities and genotoxicity evaluations

**DOI:** 10.1186/s40360-022-00622-1

**Published:** 2022-10-26

**Authors:** Ming-Yan Yang, Ze-Yu Song, Hai-Lin Gan, Mei-Hua Zheng, Qian Liu, Xiang-Ting Meng, Tao Pan, Zhen-Yuan Li, Ruo-Xuan Peng, Ke Liu, Hua-Ying Fan

**Affiliations:** 1 School of Pharmacy, Key Laboratory of Molecular Pharmacology and Drug Evaluation, Collaborative Innovation Center of Advanced Drug Delivery System and Biotech Drugs in Universities of Shandong, Ministry of Education, Yantai University, 264005 Yantai, PR China; 2 Shandong Boyuan Biomedicine Co. Ltd, 264005 Yantai, PR China; 3 Shandong Target Drug Research Co. Ltd, 264005 Yantai, PR China; 4 School of Pharmacy, Yantai University, No. 32 Qingquan Road, Laishan District, 264005 Yantai, Shandong Province China

**Keywords:** Salvianolic acid A, Acute toxicity, Subchronic toxicity, Genotoxicity, Ames test, Micronucleus

## Abstract

**Background:**

Toxicological problem associated with herbal medicine is a significant public health problem. Hence, it is necessary to elaborate on the safety of herbal medicine. Salvianolic acid A (SAA) is a major active compound isolated from Danshen, a popular herbal drug and medicinal food plant in China. The aim of the present study was to explore the toxicological profile of SAA.

**Methods:**

The acute toxicity studies were performed in mice and Beagle dogs with single administration with SAA. A 4-week subchronic toxicity was test in dogs. SAA was intravenously administered at doses of 20, 80 and 300 mg/kg. Clinical observation, laboratory testing and necropsy and histopathological examination were performed. The genotoxic potential of SAA was evaluated by 2 types of genotoxicity tests: a reverse mutation test in bacteria and bone marrow micronucleus test in mice.

**Results:**

In acute toxicities, the LD50 of SAA is 1161.2 mg/kg in mice. The minimum lethal dose (MLD) and maximal non-lethal dose (MNLD) of SAA were 682 mg/kg and 455 mg/kg in dogs, respectively. The approximate lethal dose range was 455–682 mg/kg. In the study of 4-week repeated-dose toxicity in dogs, focal necrosis in liver and renal tubular epithelial cell, the decrease in relative thymus weight, as well as abnormal changes in biochemical parameters, were observed in SAA 80 or 300 mg/kg group. The no observed adverse effect level (NOAEL) of SAA was 20 mg/kg. Thymus, liver and kidneys were the toxic targets. These toxic effects were transient and reversible. These results indicated that it should note examination of liver and kidney function during the administration of SAA in clinic. Furthermore, SAA had no mutagenic effect at any tested doses.

**Conclusion:**

These results provide new toxicological information of SAA for its clinical application and functional food consumption.

**Supplementary information:**

The online version contains supplementary material available at 10.1186/s40360-022-00622-1.

## Introduction

Traditional herbal medicine has been used worldwide. In most people’s perception, herbal medicine is low in toxicity or even non-toxic. Thus, consumers usually disregard any association between their use and any adverse reactions [1]. Toxicological problems due to the use of herbal medicine have constantly been a matter of concern to human or health care professionals. Certain of herbal medicine products associated with a serious adverse events, such as liver toxicity induced by *Lycopodium serratum*, *Lilium brownie*, *Atractylis gummifera* and *Tinospora crispa* [2, 3], nephrotoxicity induced by Chinese herbal medicine *Ephedra sinica* (ephedra/ma huang) [4] and *Ginkgo biloba* implicated in neurotoxicity [5], which seriously threaten public health [1]. However, herbal medicine has relative complex active ingredients, and there is a paucity of scientific data on their adverse effects. Hence, it is necessary to research and evaluate the toxicity of herbal medicines and their components.

Danshen, the dried root of *Salvia miltiorrhizae* Bunge (Labiatae), is one of the most versatile Chinese herbal drugs and a popular medicinal food plant in China. Danshen possesses the characteristics of facilitating blood circulation and dispersing blood stasis, and has been used for thousands of years [6]. The modern formulations of Danshen containing Danshen injection and Danshen dropping pills are commonly used clinically for the treatment of coronary heart disease, angina pectoris and myocardial injury. Their application mainly based on traditional medication experiences and knowledges. The toxicity of Danshen was sporadically recorded in ancient documents, but was not elaborated.

Salvianolic acid A (SAA) is the most active ingredients among seven water-soluble compounds of Danshen [7]. Pharmacological tests have revealed that SAA possesses a variety of pharmacological activities. It was found to prevent thrombosis formation [8, 9], inhibit platelet aggregation [8, 9], ameliorate myocardial ischemic injury [10, 11], cerebral ischemic injury [12], as well as diabetic microangiopathy [13, 14]. Currently, SAA is in a phase II clinical trial for treatment of diabetic microangiopathy (CTR20210544) and in two phase I clinical trials for treatment of angina pectoris and diabetes (CTR20181023, NCT03908242). Although there have been substantial reports concerning the pharmacological activity of SAA, there is few report about its toxicity. The objective of the present study was to assess the toxicity and safety of SAA, in the hope of providing scientific data for its clinical application and development as functional food.

## Materials and methods

### Materials

**Fig. 1:**
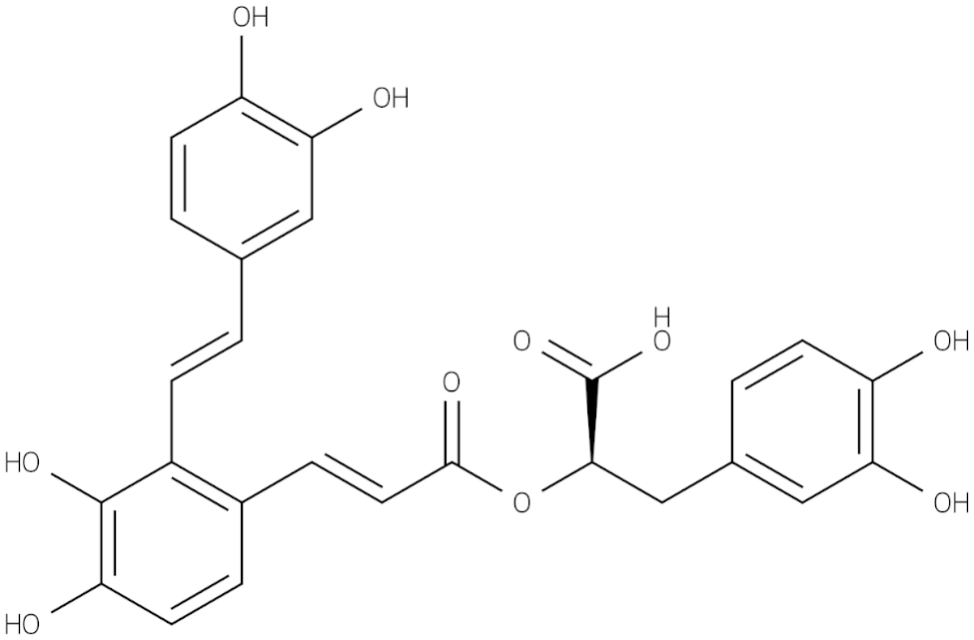
Chemical structure of Salvianolic acid A (SAA).

SAA (the chemical structure is presented in Fig. 1) was provided by Shandong Target Drug Research Co. Ltd (Shandong, China). The natural extracted SAA is low in content. Thus, SAA was synthetized through chemical conversion of raw material salvianolic acid B. This method could be scaled up for large-scale industrial production. The purity of SAA was determined to be higher than 98% by HPLC method with external standard. In this study, SAA was dissolved in 5% glucose solution.

### Experimental animals

BALB/c mice (6–7 weeks old) were provided by Experimental Animal Center of Shandong University (Jinan, China). Beagle dogs (6–9 months old) were purchased from Guangzhou Institute of Pharmaceutical Industry. All animals were acclimated for at least 1 week at a temperature of 22 ± 2℃ and relative humidity of 42%~68%. All animals were housed in cages with food and tap water ad libitum. Room were well-lit and ventilated. All experiments were performed according to the guidelines specified in the Good Laboratory Practice Regulations by China Food and Drug Administration (CFDA) and recommendations of the National Institutes of Health Guide regarding the Care and Use of Laboratory Animals. The permission of animal use was approved by Office of Experimental Animal Management Committee of Shandong Province, China (License number: SYXK [Lu] 20,180,028). The Animal Ethics Committee of Yantai University gave consent to all animal protocols.

### Acute toxicity studies in mice and dogs

A simple randomization was performed by Excel software. BALB/c mice (60 females, 60 males) were randomly assigned into the following groups (n = 20): Control, SAA (1600, 1340, 1130, 950, 800 mg/kg). After assigned to groups, mice were administered intravenously with different doses of SAA. For mice in control group, mice were administered with 5% glucose injection with an equal volume.

Six Beagle dogs (3 females, 3 males) were used in this study with approximate lethal dose method [15]. The effective dose of SAA was 10 mg/kg in rat [8, 16]. Thus, the equivalent dose of SAA in dog was 3.3 mg/kg according to the conversion of body surface area. We selected 60 mg/kg as initial dose, approximately 18 times higher than the effective dose. Successively, SAA was administered at doses of 90, 135, 202, 303, 455, 682, 1023, 1535 mg/kg, with an increasing proportion of 50%. After administration, animals were observed for changes in clinical signs and body weight. All animals were continuously observed for 14 days after administration. Finally, gross anatomy observation was carried out on dead and surviving animals at the end of the experiment. Organs with obvious pathological changes were examined histopathologically. During the experiment, the animal care staff and those who administer treatments were blind to the group allocation.

### Subchronic toxicity study

#### Study design

This work was conducted according to the “Technical guidelines of chronic toxicity for traditional Chinese medicine and natural drugs” (SFDA, 2005) [17]. A simple randomization was performed by Excel software. In brief, twenty-four Beagle dogs (12 female, 12 males) were randomly divided into four groups: Control, SAA 20, 80 and 300 mg/kg, with 6 dogs in each group. Animals were infused with SAA, once-daily for a whole month. At the end of administration, 16 dogs (2 dogs/sex/group) were sacrificed, and after the 2-week recovery period, autopsy were conducted on the remaining dogs (1 dog/sex/group) to examine delayed occurrence, persistence, and whether the toxicities were reversible. During the experiment, animal care staff and those who administer treatments were blind to group allocation.

#### Clinical observations

Animals were observed for changes in clinical signs, toxic reaction and mortality. Body weight was measured once a week, while food intake was determined every day. In addition, the body temperature measurement, ophthalmic examinations, urinalysis and electrocardiographic examination were carried out before administration, days 15 and 29 after the administration and recovery period, respectively.

#### Laboratory testing

Blood was collected for hematology and serum biochemistry analyses from the forelimb veins of animals. Hematological parameters and biochemical parameters were consistent with previous reports [18, 19].

#### Necropsy and histopathology

On days 29 and following the 2-week recovery phase, a total of 4 dogs(2 dogs/sex/group)in each group and the remaining animals in recovery period were sacrificed and dissected. Firstly, visual observation was performed, and then, absolute and relative organ weights were determined for key organs including brain, heart, liver, lung, kidneys, adrenals, thymus, spleen, testes, epididymis, uterus and ovaries. Other organs of the following were collected: spinal cord(cervical, thoracic and lumbar), pituitary, thyroid, parathyroid glands, esophagus, salivary glands, stomach, Small and large intestine, gallbladder, pancreas, trachea, aorta, prostate, mammary gland, sciatic nerve, bladder, optic nerve, bone marrow, muscle, thyroid (including parathyroid gland), lymph gland, local tissues of administration and other organs with obviously abnormal lesions. All samples were fixed in 4% neutral buffered formalin, paraffin embedded, and were sliced into 5 μm sections. Sections were stained with H&E and followed microscopic examination.

### Genotoxicity studies

#### Ames test

*Salmonella typhimurium* TA97, TA98, TA100, TA102 and TA1535 were used in this study. In a pilot study, we determined the bacterial toxicity of SAA (5000, 2500, 1250, 625, 312 µg/plate) in TA100 without a metabolic activation system. The result indicated that SAA did not show any toxicity against TA100. Thus, SAA 5000 µg/plate was selected as the maximum test concentration, other concentrations were set at 1000, 100, 10 and 1.0 µg/plate. This test was conducted by using the plate incorporation approach under the condition of presence or absence of S9 metabolic activation. We added 0.1 ml of bacterial culture solution, 0.1 ml of test article, and 0.5 ml of S9 mixture solution (metabolic activation group) into a 2 ml top culture medium in order, quickly mixed them on the oscillating mixer. Mixtures with or without S9 was plated onto basal culture medium and cultured for 48 and 72 h at 37 ℃. Positive controls included 9-aminoacridine, 2,7-diaminofluorene, sodium azide, mitomycin C, 2-aminofluorene, 1,8-dihydroxyanthraquinone. Each plate was counted for the number of revertant colonies.

#### In vivo bone marrow micronucleus assay

In vivo bone marrow micronucleus assay was conducted according to the previously described methods [18]. In brief, 60 BALB/C mice were randomly assigned into the following five groups: Vehicle control, positive control (cyclophosphamide, CP, 50 mg/kg, ip), SAA (200, 400, 800 mg/kg, iv). Mice were euthanized 24 and 48 h after administration. The sternum marrow of mice was flushed out and smears were prepared for Giemsa staining. A total of at least 2000 polychromatic erythrocytes (PCEs) were observed for the presence of micronucleus in each sample and the frequency of micronucleus in PCEs was calculated. In addition, a total of 200 erythrocytes [PCEs + normochromatic erythrocytes (NCEs)] were counted per animals. The ratio of PCEs to total erythrocytes was also calculated.

### Statistical analysis

The statistical analyses were performed using SPSS 11.0. All results were expressed as mean ± standard deviation (SD). Quantitative data were tested for homogeneity of variance. If the variance was homogeneous, one-way ANOVA followed by Dunnett test was used. Comparisons between groups of nonparametric data were made using the Kruskal-Wallis test followed by the Mann-Whitney U test. *P* < 0.05 was considered significant.

## Results

### Acute toxicity study in mice

No animals died in SAA 800 mg/kg. Four animals (4/20), 9 animals (9/20), 15 animals (15/20) and 19 animals (19/20) died in SAA 950, 1130, 1340, and 1600 mg/kg, respectively. Animal died within 30 min after administration. Dose-dependent abnormalities included decreased spontaneous motor activity, shaking head, jumping, convulsion and agony. These symptoms basically recovered 1 h after administration. Afterward, there was no longer any death. The median lethal dose (LD50) for intravenous SAA was 1161.2 mg/kg in both sexes. There were no abnormal macroscopic discoveries in surviving animals by the end of study.

### Acute toxicity study in dogs

The minimum lethal dose (MLD) and maximal non-lethal dose (MNLD) for SAA were 682 mg/kg and 455 mg/kg, respectively. The approximate lethal dose range was 455–682 mg/kg. Animals salivated during administration and showed decreased spontaneous motor activity immediately after dosing in SAA 300 mg/kg. The toxicological symptoms were comprised of abnormal gait, foam at mouth, pale tongue and diarrhea, prostration, incontinence and screaming, and decreased spontaneous motor activity, which persisted for 4–6 h after dosing until the animal died. No apparent abnormal alterations were detected at a dose of lower than 303 mg/kg. No unusual findings were evident for dead animals upon gross anatomy observation. Vacuolar degeneration of liver cells, necrosis of renal glomerulus and tubular epithelial cells, and a large amount of flocculant in renal tubules could be seen in pathological examination. No dose-related pathological changes in other organs were noted.

### Subchronic toxicity study in dogs

#### Clinical observations

**Fig. 2:**
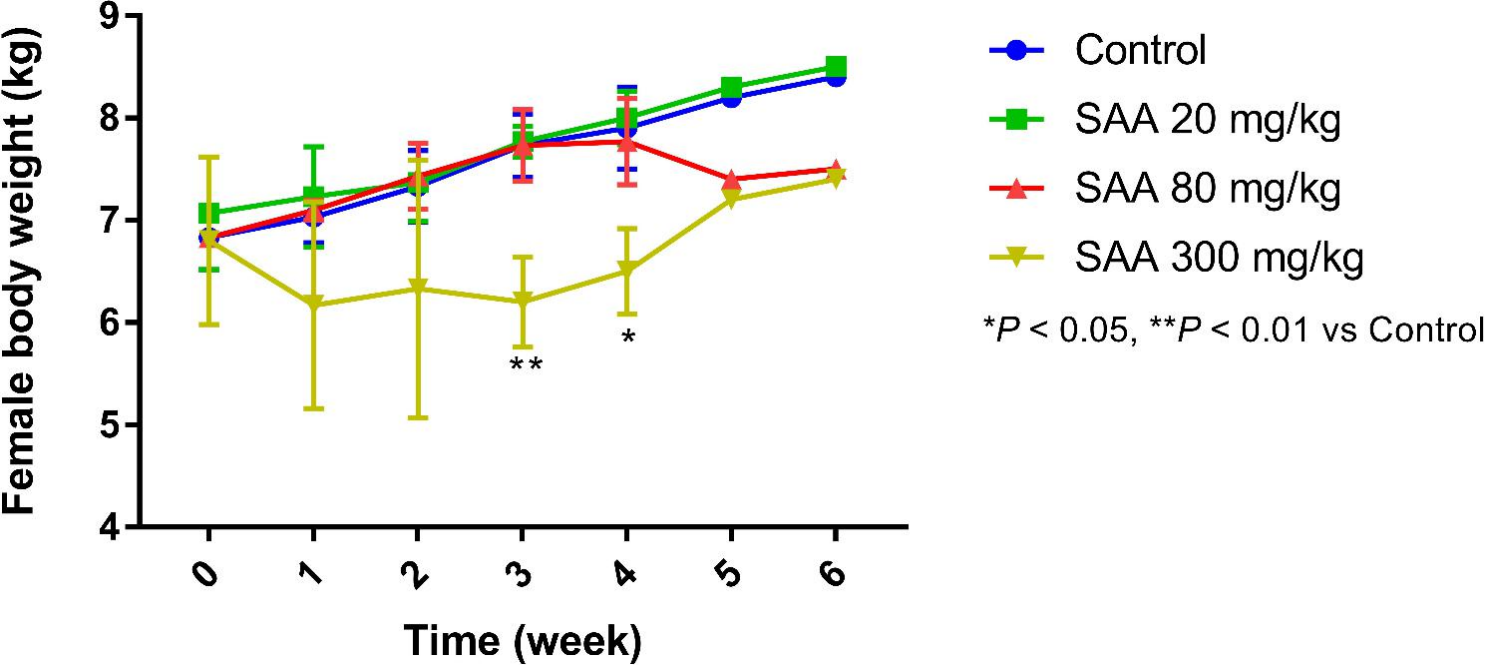
Changes in body weight in female dogs during SAA administration period (n = 3) and following a 2-week recovery period (n = 1). After a 4-week treatment period, drug treatment was discontinued and animals were allowed to recover for 2 weeks

**Fig. 3:**
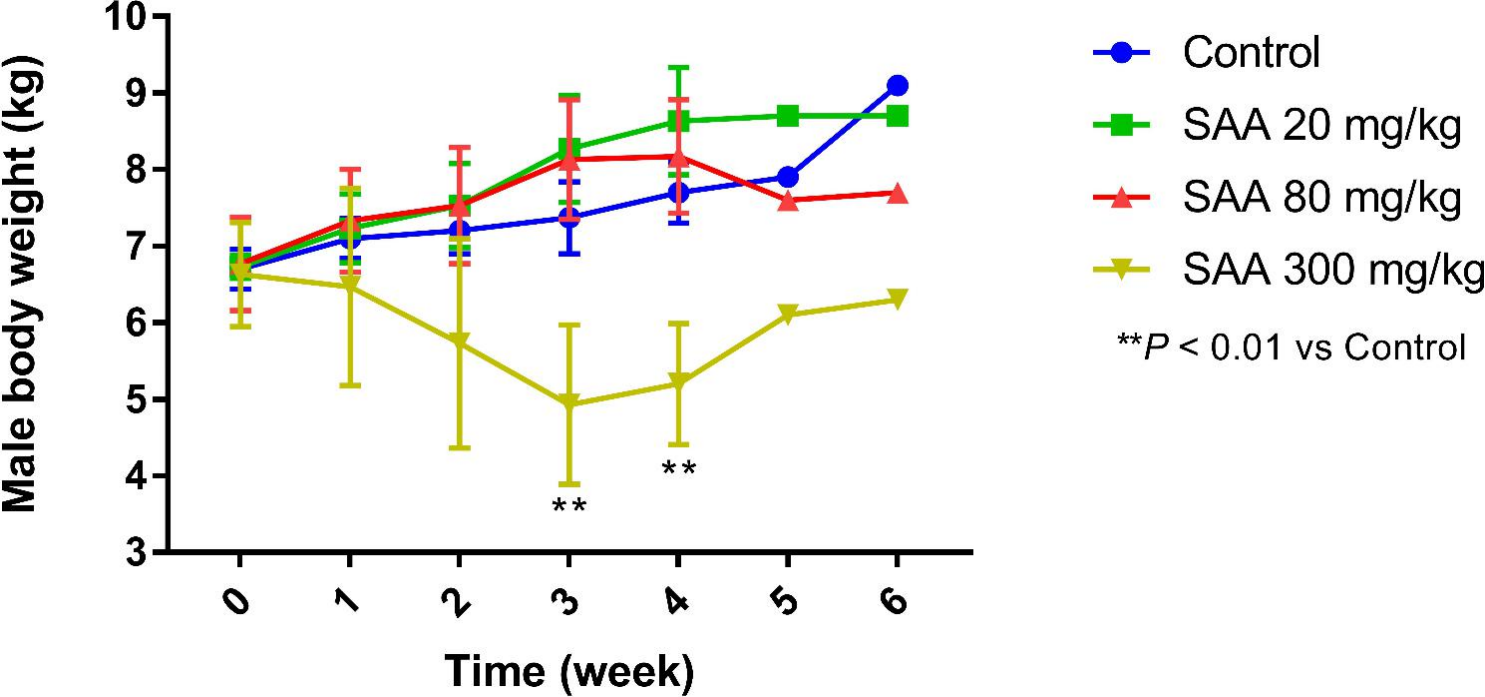
Changes in body weight in male dogs during SAA administration period (n = 3) and following a 2-week recovery period (n = 1). After a 4-week treatment period, drug treatment was discontinued and animals were allowed to recover for 2 weeks

There were no clinical abnormalities in animals treated with SAA 20 and 80 mg/kg. Animals salivated and had decreased or even lost spontaneous motor activity after dosing in SAA 300 mg/kg group. Very few animal vomited. All abnormalities described above dissolved within 2 h after dosing. On days 20, there was a female animal near to death in the group of SAA 300 mg/kg, and then the animal was dissected for examination. No obvious differences were documented in all groups following recovery period. As depicted in Figs. 2 and 3, the body weight did not differ significantly in SAA (20, 80 mg/kg) and control groups, which was lower in SAA 300 mg/kg than that in other groups, with statistically significant difference in contrast to control at 3- and 4-week after administration (*P* < 0.05 or *P* < 0.01). During the recovery period, body weight were comparable in control and SAA (Figs. 2 and 3). Likewise, the food intake was decreased in SAA 300 mg/kg on days 2 after dosing, which gradually returned to normal in the later stage of experiment. Furthermore, ophthalmic examination, body temperature and urinalysis showed no obvious discrepancy in all groups (data not shown).

#### Hematological findings and blood chemistry

As compared with control, hemoglobin (HGB) and hematocrit (HCT) levels markedly decreased in SAA 300 mg/kg (*P* < 0.01) (Table 1). Similarly, the levels of total bilirubin (T-BIL), blood urea nitrogen (BUN), triglyceride (TG) significantly elevated, and K^+^ and Na^+^ levels lowered (Table 2). No obvious differences were observed in other parameters. At the end of recovery period, all parameters, except TG, did not significantly differ in animals in SAA and control group. The level of TG rose in all SAA-treated groups, but not in a dose-dependent manner (Table 2).

**Table 1:** Effects of SAA on Hematology of dogs

Parameter Detection time	Control	SAA 20 mg/kg	SAA 80 mg/kg	SAA 300 mg/kg
WBC (10^9^/L) End of dose phase End of recovery phase	14.11 ± 2.48 12.93 ± 5.30	11.59 ± 1.89 12.24 ± 1.22	10.99 ± 2.02 10.57 ± 3.15	14.19 ± 8.29 10.73 ± 0.89
NEU % (%) End of dose phase End of recovery phase	76.72 ± 10.06 67.24 ± 7.54	74.30 ± 7.58 68.23 ± 5.64	72.70 ± 6.13 72.59 ± 3.49	81.88 ± 4.37 84.60 ± 6.01
LYM% (%) End of dose phase End of recovery phase	12.34 ± 5.99 16.55 ± 5.02	15.01 ± 4.70 19.23 ± 8.34	15.74 ± 4.57 15.26 ± 2.35	10.82 ± 3.64 7.57 ± 1.28
MONO% (%) End of dose phase End of recovery phase	7.24 ± 3.20 10.86 ± 5.65	7.11 ± 1.97 8.28 ± 0.83	7.65 ± 2.92 8.10 ± 0.82	5.31 ± 1.88 6.53 ± 4.57
EOS% (%) End of dose phase End of recovery phase	3.31 ± 1.40 5.07 ± 2.91	3.01 ± 1.86 3.86 ± 3.74	3.57 ± 1.22 3.57 ± 0.29	1.78 ± 0.98 1.09 ± 0.18
BASO% (%) End of dose phase End of recovery phase	0.38 ± 0.35 0.29 ± 0.21	0.57 ± 0.40 0.43 ± 0.21	0.35 ± 0.51 0.48 ± 0.04	0.20 ± 0.11 0.21 ± 0.01
RBC (10^12^/L) End of dose phase End of recovery phase	6.73 ± 0.40 6.38 ± 0.15	7.09 ± 0.52 6.73 ± 0.43	6.35 ± 0.38 6.74 ± 0.53	6.11 ± 0.90 5.15 ± 0.36
HGB (g/dL) End of dose phase End of recovery phase	15.22 ± 1.44 14.15 ± 1.20	15.95 ± 1.36 15.60 ± 0.28	14.38 ± 0.50 15.60 ± 1.27	12.84 ± 1.80* 11.45 ± 1.63
HCT (fL) End of dose phase End of recovery phase	46.62 ± 3.89 42.70 ± 4.53	49.02 ± 3.37 46.80 ± 1.56	44.45 ± 1.59 46.90 ± 2.83	39.26 ± 5.49** 35.25 ± 5.44
MCV (fL) End of dose phase End of recovery phase	69.25 ± 3.48 66.95 ± 5.59	69.18 ± 1.77 69.70 ± 2.12	70.22 ± 3.58 69.70 ± 1.27	64.50 ± 5.35 68.30 ± 5.80
MCH (pg) End of dose phase End of recovery phase	22.60 ± 1.33 22.15 ± 1.34	22.50 ± 0.78 23.25 ± 1.06	22.75 ± 1.34 23.15 ± 0.07	21.10 ± 1.43 22.20 ± 1.56
MCHC (g/dL) End of dose phase End of recovery phase	32.63 ± 0.77 33.20 ± 0.71	32.53 ± 1.05 33.35 ± 0.49	32.37 ± 0.61 33.20 ± 0.71	32.76 ± 1.01 32.50 ± 0.42
PLT (10^9^/L) End of dose phase End of recovery phase	378.17 ± 59.50 273.00 ± 43.84	319.17 ± 43.21 337.50 ± 89.80	307.67 ± 61.31 288.50 ± 4.95	312.40 ± 128.53 456.00 ± 117.38
RET% (%) End of dose phase End of recovery phase	11.73 ± 7.34 15.10 ± 8.77	8.63 ± 0.48 7.90 ± 0.28	8.93 ± 0.70 8.40 ± 0.42	8.70 ± 0.71 8.50 ± 0.28
PT (sec) End of dose phase End of recovery phase	1.48 ± 0.12 1.50 ± 0.14	1.38 ± 0.23 1.60 ± 0.14	1.47 ± 0.27 1.60 ± 0.28	1.48 ± 0.33 1.55 ± 0.35
*P < 0.05, **P < 0.01 vs. Control. Abbreviations: WBC, White blood cell count; NEU, Neutrophil; LYM, Lymphocyte; MONO, Monocyte; EOS, Eosinophils; BASO, Basophil; RBC, Red blood cell; HGB, Hemoglobin; HCT, Hematocrit; MCV, Mean corpuscular volume; MCH, Mean corpuscular hemoglobin; MCHC, Mean corpuscular hemoglobin concentration; PLT, Platelet; RET, Reticulocyte; PT, Thrombin time.				

**Table 2:** Effects of SAA on clinical chemistry of dogs

Parameter Detection time	Control	SAA 20 mg/kg	SAA 80 mg/kg	SAA 300 mg/kg
AST (U/L) End of dose phase End of recovery phase	40.33 ± 6.06 47.00 ± 1.41	44.50 ± 7.40 36.50 ± 6.36	40.50 ± 8.22 39.50 ± 4.95	49.40 ± 21.31 39.50 ± 3.54
ALT (U/L) End of dose phase End of recovery phase	29.17 ± 6.43 27.00 ± 1.41	32.67 ± 5.72 21.00 ± 2.83	47.17 ± 15.96 23.00 ± 11.31	35.80 ± 19.99 20.00 ± 4.24
ALP (U/L) End of dose phase End of recovery phase	74.87 ± 30.89 56.30 ± 1.84	71.90 ± 16.09 57.65 ± 2.05	80.18 ± 21.62 53.05 ± 19.59	64.44 ± 15.98 47.70 ± 4.10
T-BIL(µmol/L) End of dose phase End of recovery phase	0.64 ± 0.41 0.48 ± 0.04	0.86 ± 0.40 0.59 ± 0.12	2.25 ± 1.29 0.55 ± 0.03	10.59 ± 13.05* 0.86 ± 0.30
BUN (mmol/L) End of dose phase End of recovery phase	4.86 ± 0.78 4.07 ± 0.77	4.98 ± 0.64 4.44 ± 1.12	5.04 ± 0.66 4.66 ± 0.07	6.97 ± 2.42* 6.61 ± 2.96
CRE (µmol/L) End of dose phase End of recovery phase	52.48 ± 5.91 52.30 ± 5.94	52.65 ± 5.57 56.20 ± 3.96	55.52 ± 6.50 56.00 ± 9.19	46.34 ± 9.56 50.90 ± 8.77
TP (g/L) End of dose phase End of recovery phase	62.07 ± 5.69 63.00 ± 5.23	60.37 ± 3.81 60.70 ± 0.85	60.40 ± 6.14 67.70 ± 0.42	58.40 ± 8.32 59.35 ± 10.82
ALB (g/L) End of dose phase End of recovery phase	29.20 ± 3.98 32.75 ± 2.19	31.70 ± 1.55 33.60 ± 4.24	29.20 ± 2.30 32.60 ± 1.41	26.02 ± 4.70 30.70 ± 6.79
CHOL (mmol/L) End of dose phase End of recovery phase	3.88 ± 0.56 4.24 ± 0.31	4.28 ± 0.33 4.61 ± 0.25	4.28 ± 0.33 5.11 ± 1.04	4.33 ± 0.50 4.83 ± 0.49
TG (mmol/L) End of dose phase End of recovery phase	0.38 ± 0.16 0.59 ± 0.08	0.47 ± 0.15 0.78 ± 0.01*	0.48 ± 0.12 0.78 ± 0.05*	0.70 ± 0.27* 0.77 ± 0.03*
GLU (mmol/L) End of dose phase End of recovery phase	4.90 ± 0.21 6.68 ± 0.28	5.49 ± 0.37 7.12 ± 0.28	5.50 ± 0.33 6.36 ± 0.33	5.85 ± 2.17 6.89 ± 0.35
CK (U/L) End of dose phase End of recovery phase	200.83 ± 64.72 290.50 ± 10.61	212.00 ± 61.54 238.50 ± 156.27	190.50 ± 31.13 140.00 ± 12.73	250.60 ± 145.21 153.00 ± 49.50
GGT (U/L) End of dose phase End of recovery phase	2.83 ± 1.60 3.50 ± 0.71	2.67 ± 0.82 2.50 ± 0.71	2.50 ± 0.84 2.00 ± 0.00	3.00 ± 1.73 4.50 ± 3.54
K^+^ (mmol/L) End of dose phase End of recovery phase	5.02 ± 0.17 4.95 ± 0.21	4.80 ± 0.06 4.75 ± 0.21	4.63 ± 0.10 4.90 ± 0.14	4.24 ± 0.61** 4.95 ± 0.07
Na^+^ (mmol/L) End of dose phase End of recovery phase	147.67 ± 0.82 148.50 ± 0.71	148.00 ± 1.26 147.50 ± 0.71	147.50 ± 0.84 149.00 ± 1.41	142.60 ± 6.47** 150.50 ± 2.12
Cl^−^ (mmol/L) End of dose phase End of recovery phase	112.00 ± 0.89 113.00 ± 1.41	112.17 ± 1.83 113.00 ± 0.00	112.00 ± 1.41 115.50 ± 0.71	104.20 ± 12.52* 113.50 ± 0.71
*P < 0.05, **P < 0.01 vs. Control. Abbreviations: AST, Aspartate aminotransferase; ALT, Alanine aminotransferase; ALP, Alkaline phosphatase; T-BIL, Total bilirubin; BUN, Blood urea nitrogen; CRE, Creatinine; TP, Total protein; ALB, Albumin; CHOL, Cholesterol; TG, Triglyceride; GLU, Glucose; CK, Creatine kinase; GCT, Glutamyl transpeptidase.				

#### Pathological findings

No macroscopic differences were observed at the end of dosing and recovery. The relative thymus weight obviously reduced in animals in SAA 300 mg/kg, which was still lower in male animals than that in control following recovery period. No significant abnormalities were found in other relative organ weights (Supplementary Table S1 and S2).

**Fig. 4:**
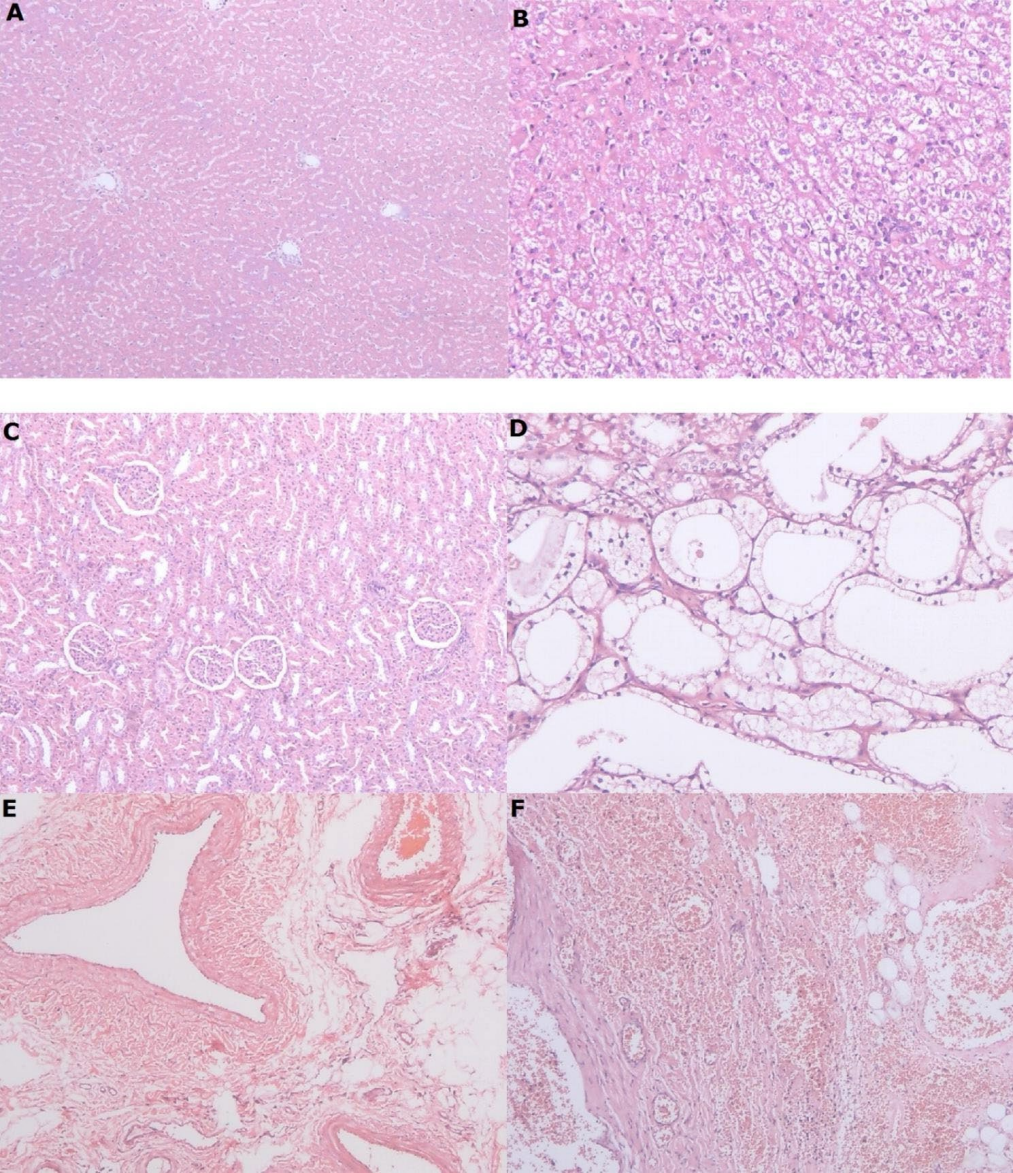
Representative images of pathological changes showed vacuolated hepatocytes with necrosis in liver (B), necrosis in renal tubular epithelial cell (D) (10×magnification ) and haemorrhage and inflammatory cell infiltration in local tissue of administration (F, 5×magnification ) compared with normal control (A, C and E, 5×magnification)

In the histopathological examination, haemorrhage and inflammatory cell infiltration in local tissue of administration were found in 1 animal in control (1/4) and 2 animals in SAA 20 mg/kg (2/4). Focal necrosis in liver (2/4), and haemorrhage and inflammatory cell infiltration in local tissue of administration (2/4) were found in SAA 80 mg/kg. Vacuolated hepatocytes with necrosis in liver (3/4), necrosis in renal tubular epithelial cell (3/4) and haemorrhage and inflammatory cell infiltration in local tissue of administration (2/4) were observed in SAA 300 mg/kg (Fig. 4). Thymus and other organs had no dose-related pathological alterations. After 2-week recovery periods, necrosis in renal tubular epithelial cell (1/2) and renal tubular vacuolization (1/2) were still existed in animals treated with SAA 300 mg/kg (data not shown). Pathological changes observed in other groups had been recovered.

### Ames test

The number of revertant colonies of each test strain in the vehicle control all fall within the normal range. As shown in Table 3, the revertant colonies counts in the positive control significantly higher than that in the vehicle control. SAA at doses of 5000, 1000, 100, 10 and 1 µg/plate did not elevated the number of revertant colonies, either with or without S9, which indicated that SAA had no inducing effect on gene mutation of histidine deficient *Salmonella typhimurium* (Table 3).

**Table 3:** Effect of SAA on bacterial mutation assay

Test Article	Dose (µg/plate)	S9 mix	Number of revertant colonies				
			TA97	TA98	TA100	TA1535	TA102
Vehicle control		-	145.0 ± 2.0	38.7 ± 4.5	151.7 ± 9.9	19.0 ± 7.5	252.0 ± 23.5
		+	128.0 ± 6.0	40.0 ± 20.7	141.0 ± 25.1	16.0 ± 6.9	287.0 ± 22.1
Positive control		-	1508.0 ± 10.6①	247.7 ± 25.5②	3821.3 ± 398.5③	2288.0 ± 246.6③	986.7 ± 282.3④
		+	1228.0 ± 25.0⑤	404.0 ± 43.3⑤	2612.0 ± 255.9⑤	1729.3 ± 93.6⑤	1218.3 ± 149.1⑥
SAA	5000	-	125.3 ± 20.6	28.3 ± 2.1	159.0 ± 14.9	16.7 ± 4.5	246.0 ± 17.6
		+	146.3 ± 3.1	42.0 ± 13.9	155.0 ± 11.1	14.3 ± 2.9	304.7 ± 17.4
	1000	-	143.0 ± 6.0	31.7 ± 4.9	162.3 ± 23.2	15.3 ± 4.9	251.3 ± 20.6
		+	152.0 ± 18.0	38.7 ± 6.7	161.0 ± 4.4	18.7 ± 0.6	298.0 ± 24.0
	100	-	136.7 ± 8.5	35.0 ± 7.8	160.7 ± 11.0	11.7 ± 2.5	250.7 ± 13.2
		+	125.0 ± 33.1	45.0 ± 16.8	148.0 ± 2.6	14.7 ± 4.2	295.0 ± 21.7
	10	-	135.3 ± 10.4	34.7 ± 4.0	168.3 ± 6.4	12.3 ± 4.0	238.3 ± 6.7
		+	140.0 ± 9.0	43.0 ± 15.6	142.7 ± 3.8	16.3 ± 3.8	298.7 ± 27.4
	1	-	137.7 ± 8.5	40.0 ± 2.6	153.3 ± 2.1	17.7 ± 1.5	254.0 ± 5.2
		+	139.0 ± 10.6	46.3 ± 11.7	129.0 ± 21.0	16.7 ± 4.5	287.7 ± 13.6
①9-aminoacridine 50 µg/plate ② 2,7-diaminofluorene 20 µg/plate ③Sodium azide 1.5 µg/plate ④Mitomycin C 0.5 µg/plate ⑤2-aminofluorene 50 µg/plate ⑥1,8-dihydroxyanthraquinone 50 µg/plate							

### In vivo murine bone marrow micronucleus assay

No significant differences were observed in the micronucleus formation rate and the ratio of PCEs to the total erythrocytes in animals treated with SAA (50-800 mg/kg) as compared with that of vehicle control at 24 or 48 h after administration (Table 4).

**Table 4:** Effect of SAA on micronucleus formation

Group	Time of post-administration	Dose (mg/kg)	Micronucleus	MNPCE (‰)	PCE/(NCE + PCE)
Control	24 h	/	30	2.50	0.614 ± 0.026
CP		50	303	25.25**	0.386 ± 0.014**
SAA		200	26	2.17	0.614 ± 0.021
		400	20	1.67	0.598 ± 0.035
		800	20	1.67	0.596 ± 0.037
Control	48 h	/	25	2.08	0.608 ± 0.034
CP		50	282	23.50**	0.385 ± 0.015**
SAA		200	19	1.58	0.595 ± 0.031
		400	19	1.58	0.594 ± 0.022
		800	21	1.75	0.605 ± 0.032
**P < 0.01 vs. Control. Abbreviations: CP, Cyclophosphamide.					

## Discussion

Salvianolic acid A possesses important biological activities like anti-thrombosis, anti-oxidant, improving learning and memory, which is more representative of the activities of facilitating blood circulation and dispersing blood stasis of Danshen in contrast to early discovered diterpenoids. In recent years, SAA has drawn great interest for its diverse potent bioactivities. However, there is insufficient security data of SAA to guide its usage in human. Thus, we evaluated its toxicological profile in the present study.

As compared with other traditional herbal medicines, such as *Ephedra sinica* (ephedra/ma huang), *Tripterygium wilfordii* and *Ginkgo biloba*, Danshen has better security. Previously studies have reported the toxicity of Danshen injection in rats and Beagle’s dogs [20, 21]. The results demonstrated that Danshen injection was low-toxic or non-toxic. The major adverse effects were changes in hematological and biochemical parameters, as well as the lesions of the injection site. Thus, as a major active constitute of Danshen, we speculated that SAA possessed a good safety profile as well.

In the acute toxicity studies, SAA showed no severe toxic responses. The LD50 of SAA was 1161.2 mg/kg in mice with single intravenous dose. No abnormal pathological changes were observed. The MLD and MNLD were 682 and 455 mg/kg for SAA in dogs with single intravenous administration. The pathological changes were noted in liver and kidneys, which indicted that liver and kidneys may be the toxic target organs of SAA in chronic toxicity study.

To further verify the repeated dose toxicity of SAA, we conducted a 4-week subchronic toxicity test in dogs. During the experiment, daily treatment with SAA for 4 weeks had no obvious effect on ophthalmic examination, body temperature, urinalysis as well as electrocardiogram (ECG) examination in dogs. SAA at dose of 300 mg/kg markedly affected body weight gain of dogs, accompanied by obviously decreased in food intake.

The decreases in HGB and HCT levels were only found in SAA 300 mg/kg, which recovered to normal range following recovery period. Although it had no obvious time-response relationship, we cannot ruled out that the abnormal changes were caused by administration. In addition, the increases in levels of T-BIL, BUN and TG, and decreases in levels of K^+^, Na^+^, Cl^−^, were found in SAA 300 mg/kg. According to the result of verification testing, SAA had greater impact in T-BIL determination when added into blank serum. Thus, the phenomenon that T-BIL level increased dozens of times in SAA 300 mg/kg was directly caused by SAA, not toxicological manifestation. Although TG level increased, the value still fall within the normal range. Following the recovery period, TG levels equally increased in all groups treated with SAA. This change was not dose-related, and the values also fall within the normal range. We therefore believed that this change was the normal physiological fluctuation of animals and had no toxicological significance. The result of increase in BUN level was consistent with that of renal injury caused by SAA, so it can be considered as an outcome of treatment with SAA. The phenomenon of decrease in levels of K^+^, Na^+^ and Cl^−^ can be speculated that SAA can caused the change of iron level in vivo.

Then again, we found that thymus weight and relative thymus weight decreased after treatment with higher dosage of SAA. Although there was no dose-related pathological changes to be found, this change may be an abnormal response induced by long-term treatment with SAA, that is, thymus may be a toxic target organ of SAA.

The histological changes were found in liver and kidney, which mainly manifested as focal necrosis in liver and renal tubular epithelial cell, vacuolated hepatocytes with necrosis in liver and renal tubular vacuolization. Hepatotoxicity occurred both in SAA 80 and 300 mg/kg, while nephropathy mainly occurred in SAA 300 mg/kg. These pathological changes were obviously time-effect related. These evidences of toxicities indicating that liver and kidney were another possible toxic target organs of SAA. After 2-week recovery period, hepatic lesions were not found in both groups, and nephropathy basically recovered, further indicating that the toxicity of SAA were reversible. In our previous study in rats, the pharmacokinetics of SAA followed a two-compartment model, with a rapid distribution and an elimination with a half-life of approximately 1 h. Intravenous administration of SAA was rapidly distributed in all tissues, with the highest levels occurring in the heart, liver, kidney and lung (data not shown). The result was consistent with that of toxicity study in dog, further supporting the liver and kidney as toxic target organs of SAA. In vivo, SAA is metabolized and eliminated primarily as conjugated metabolites, and bile acid is the main metabolic pathway [22]. All these indicated that the examination of liver and kidney functions should be noted during the administration of SAA in clinic. The presence of hepatobiliary injury should be noted to prevent drug accumulation.

Moreover, pathological lesions were noted in local tissues of administration in animal of all groups, manifested as haemorrhage and inflammatory cell infiltration. After 2-week recovery period, no abnormalities were found in all groups. Due to the lesions were transient and not dose-related, we considered that the histopathological changes were caused by mechanical stimulation during administration, and were not dose-related toxic effect.

The genotoxic potential of SAA was evaluated by 2 types of genotoxicity tests: a reverse mutation test in bacteria and bone marrow micronucleus test in mice. In a reverse mutation assay using five strains of *Salmonella typhimurium*, SAA (1, 10, 100, 1000, and 5000 µg/plate) did not elevate the amount of revertant colonies in any tester strain, with or without S9 metabolic activation. In an in vivo bone marrow micronucleus test, mice were treated intravenously with SAA at a dose up to 5000 mg/kg. There was no significant or dose-dependent elevation in the ratios of mononuclear polychromatic erythrosite (MNPCE) (‰). The results indicated that SAA had no effect on micronucleus formation and inhibition on hematopoietic function of bone marrow cells.

## Conclusion

To sum up, in acute toxicities, the LD50 of SAA was 1161.2 mg/kg in mice. The MLD and MNLD of SAA were 682 mg/kg and 455 mg/kg in dogs, respectively. The approximate lethal dose range was 455–682 mg/kg. In the study of 4-week repeated-dose intravenous toxicity, the no observed adverse effect level (NOAEL) of SAA was 20 mg/kg in Beagle dogs following daily intravenous administration. Long-term administration of high SAA dosage can be toxic to thymus, liver and kidney. The toxic effects were transient and reversible, and no delayed onset toxicity was observed. It is indicted that if liver function, renal function and thymus function indicators are abnormal during the clinical study, the drug should be stopped or the regimen adjusted. Those with impaired liver and kidney function should take SAA cautiously and pay more attention to their health. Furthermore, SAA had no mutagenic effect at any tested doses. Taken together, these results provide new toxic information of SAA for its clinical application and functional food consumption.

## Electronic supplementary material

Below is the link to the electronic supplementary material.

Supplementary Material 1

## Data Availability

All data generated or analysed during this study are included in this published article [and its supplementary information files].

## Declarations

### Standards of reporting

The study is reported in accordance with ARRIVE guidelines.
