# Supplementary material for: Non-clinical safety evaluation of salvianolic acid A: acute, 4-week intravenous toxicities and genotoxicity evaluations
Source: BMC Pharmacol Toxicol. 2022 Oct 26;23:83. doi: 10.1186/s40360-022-00622-1 (PMC9597988; doi:10.1186/s40360-022-00622-1)
Supplement: Supplementary file 1 — Supplementary Material 1 [file 40360_2022_622_MOESM1_ESM.doc]

**Non-clinical safety evaluation of Salvianolic acid A: acute, 4-week intravenous toxicities and genotoxicity evaluations**

Ming-Yan Yang^a^, Ze-Yu Song^a^, Hai-Lin Gan^a^, Mei-Hua Zheng^a^, Qian Liu^a^, Xiang-Ting Meng^a^, Tao Pan^a^, Zhen-Yuan Li^a^, Ruo-Xuan Peng^a^, Ke Liu^b,c^, Hua-Ying Fan^a *^

**Supplementary material**

Table S1 Relative organ weight in female dogs treated with SAA (g/kg)

|  | Control | SAA 20 mg/kg | SAA 80 mg/kg | SAA 300 mg/kg |
| --- | --- | --- | --- | --- |
| **End of dose phase** |  |  |  |  |
| Heart | 6.69±1.02 | 6.73±0.63 | 6.80±0.77 | 6.87±0.91 |
| Liver | 36.03±5.13 | 34.50±0.19 | 33.07±3.51 | 35.60±7.43 |
| Spleen | 3.29±0.13 | 4.03±1.64 | 4.34±1.66 | 3.41±0.74 |
| Lung | 9.59±0.64 | 8.80±0.09 | 9.88±0.64 | 10.25±0.28 |
| Kidneys | 2.61±0.26 | 3.06±0.31 | 2.74±1.97 | 3.75±0.60 |
| Adrenals | 0.0713±0.0165 | 0.0569±0.0108 | 0.0705±0.0138 | 0.0854±0.0105 |
| Ovaries | 0.0459±0.0147 | 0.0424±0.0190 | 0.0571±0.0198 | 0.0512±0.0033 |
| Uterus | 0.15±0.05 | 0.17±0.08 | 0.15±0.01 | 0.14±0.01 |
| Thymus | 1.49±0.43 | 1.36±0.23 | 1.51±0.24 | 0.68±0.37 |
| Brain | 8.11±0.23 | 8.52±0.03 | 8.31±0.85 | 9.31±0.27 |
| **End of recovery** |  |  |  |  |
| Heart | 7.57 | 6.76 | 7.65 | 6.80 |
| Liver | 23.91 | 27.26 | 35.70 | 28.8 |
| Spleen | 2.41 | 2.57 | 2.94 | 2.42 |
| Lung | 9.22 | 6.99 | 8.83 | 10.27 |
| Kidneys | 2.43 | 2.65 | 2.78 | 3.61 |
| Adrenals | 0.0572 | 0.0871 | 0.0807 | 0.0676 |
| Ovaries | 0.0411 | 0.0441 | 0.0560 | 0.0453 |
| Uterus | 0.13 | 0.16 | 0.29 | 0.11 |
| Thymus | 1.57 | 1.35 | 0.79 | 1.10 |
| Brain | 9.71 | 8.00 | 8.23 | 8.08 |

Table S2 Relative organ weight in male dogs treated with SAA

|  | Control | SAA 20 mg/kg | SAA 80 mg/kg | SAA 300 mg/kg |
| --- | --- | --- | --- | --- |
| **End of dose phase** |  |  |  |  |
| Heart | 6.62±0.44 | 5.92±0.22 | 6.26±1.05 | 8.09±2.27 |
| Liver | 29.64±0.22 | 22.91±0.30 | 26.30±3.83 | 42.32±0.87 |
| Spleen | 3.15±0.69 | 4.41±2.85 | 2.13±0.38 | 3.27±0.30 |
| Lung | 8.59±0.61 | 9.01±0.15 | 9.11±0.45 | 12.87±3.11 |
| Kidneys | 2.81±0.10 | 2.53±0.29 | 2.71±0.07 | 6.68±3.27 |
| Adrenals | 0.0684±0.0186 | 0.0517±0.0105 | 0.0509±0.0036 | 0.1263±0.0411 |
| Testes | 0.14±0.03 | 0.16±0.10 | 0.11±0.01 | 0.13±0.02 |
| Epididymides | 0.0798±0.0132 | 0.0849±0.0121 | 0.0584±0.0118 | 0.1116±0.0075 |
| Thymus | 0.83±0.06 | 1.37±0.05 | 1.87±1.00 | 0.23±0.02 |
| Brain | 8.85±0.28 | 8.29±1.06 | 7.92±0.29 | 13.42±2.57 |
| **End of recovery** |  |  |  |  |
| Heart | 7.28 | 6.21 | 5.94 | 4.55 |
| Liver | 29.95 | 24.14 | 28.65 | 20.77 |
| Spleen | 1.96 | 2.45 | 3.02 | 2.10 |
| Lung | 9.73 | 11.90 | 8.04 | 6.94 |
| Kidneys | 3.27 | 2.55 | 3.24 | 4.28 |
| Adrenals | 0.0679 | 0.0724 | 0.0753 | 0.1024 |
| Testes | 0.0766 | 0.1569 | 0.1371 | 0.0540 |
| Epididymides | 0.0667 | 0.0701 | 0.0669 | 0.0604 |
| Thymus | 1.28 | 1.59 | 1.00 | 0.09 |
| Brain | 8.36 | 7.87 | 9.82 | 9.76 |
